# Bovine Lactoferrin Modulates Dendritic Cell Differentiation and Function

**DOI:** 10.3390/nu10070848

**Published:** 2018-06-29

**Authors:** Olaf Perdijk, R. J. Joost van Neerven, Erik van den Brink, Huub F. J. Savelkoul, Sylvia Brugman

**Affiliations:** 1 Cell Biology and Immunology Group, Wageningen University, P.O. Box 338, 6708 WD Wageningen, The Netherlands; olaf.perdijk@wur.nl (O.P.); joost.vanneerven@wur.nl (R.J.J.v.N.); erik.vandenbrink@wur.nl (E.v.d.B.); huub.savelkoul@wur.nl (H.F.J.S.); 2 FrieslandCampina, P.O. Box 1551, 3800 BN Amersfoort, The Netherlands

**Keywords:** bovine lactoferrin, moDC, DC differentiation, semi-mature phenotype, hyporesponsive, LPS

## Abstract

Lactoferrin is an abundant glycoprotein in bovine milk that has immunomodulatory effects on human cells. Bovine lactoferrin (LF) binds lipopolysaccharides (LPS) with high affinity and is postulated to act via TLR4-dependent and -independent mechanisms. It has been shown that LF modulates differentiation of human monocytes into tolerogenic dendritic cells. However, in a previous study, we showed that LPS also mediates differentiation into tolerogenic dendritic cells (DC). Since LF binds LPS with high affinity, it remains to be investigated whether LF or LPS is mediating these effects. We, therefore, further investigated the LPS-independent effect of LF on differentiation of human monocytes into dendritic cells (DC). Human monocytes were isolated by magnetic cell sorting from freshly isolated PBMCs and cultured for six days in the presence of IL-4 and GM-CSF with or without LF or proteinase K treated LF to generate DC. These immature DC were stimulated for 48 h with LPS or Poly I:C + R848. Cell surface marker expression and cytokine production were measured by flow cytometry. DC differentiated in the presence of LF produced higher IL-6 and IL-8 levels during differentiation and showed a lower expression of CD1a and HLA-DR. These LFDCs showed to be hyporesponsive towards TLR ligands as shown by their semi-mature phenotype and reduced cytokine production. The effect of LF was abrogated by proteinase K treatment, showing that the functional effects of LF were not mediated by LPS contamination. Thus, LF alters DC differentiation and dampens responsiveness towards TLR ligands. This study indicates that LF can play a role in immune homeostasis in the human GI tract.

## 1. Introduction

Bovine lactoferrin (LF) is an abundant glycoprotein in cow’s milk that is 69% identical to human lactoferrin at the protein level [1]. LF is an extensively researched protein that has been shown to exert antimicrobial and antiviral activity [2]. The involved anti-pathogenic mechanisms, which are mostly investigated in vitro, range from depriving iron, antimicrobial activity by bioactive peptides and decoy receptor activity. These mechanisms may underlie the protective effect against sepsis by LF supplementation in very low birth weight (VLBW) infants [3,4,5,6,7]. LF was shown to protect against sepsis in VLBW infants in either breast fed or formula fed, showing the need for additional supplementation [3]. Subsequent analysis of this study showed that LF inhibits the progression of invasive fungal infections [4], indicating immunomodulatory effects of LF. In line with these findings, LF was shown to induce the prevalence of regulatory T cells in VLBW infants [6]. Although these studies indicate an immunoregulatory role of LF in humans, little mechanistic evidence is available to date.

In infants, LF ends up in the intestine intact and is only partly hydrolysed into bioactive peptides with antibacterial activity [8]. Moreover, intact human lactoferrin was found in stool and urine of VLBW infants and resisted trypsin and chemotrypsin treatment in vitro [9]. LF was shown to largely resists gastric hydrolysis in vivo in adults [10]. However, LF is completely degraded in the small intestine of adults [11]. Thus LF may, in contrast to adults, retain its bioactivity throughout the gastro-intestinal tract and may even become systemically available in infants [9,12,13]. This could be explained by lower concentrations of proteases in the small intestine in infants compared to adults [14]. Additionally, breast milk contains protease inhibitors, which may limit degradation of milk proteins in the GI tract of infants [15].

LF has specific domains that bind iron with high affinity, a high isoelectric point (pI around 9) and an overall nett positive charge with high cationic peptide regions, which is crucial for its bactericidal activity [16]. Due to these biochemical properties, LF may bind multiple receptors (e.g., intelectin-1 and DC-SIGN) with low affinity [1,17,18]. LF was shown to bind the human lactoferrin receptor (i.e., intelectin-1) on Caco-2 cells and, dependent on the concentration, induce proliferation or differentiation of these epithelial cells [19].

Additionally, LF is known for its binding activity to lipopolysaccharides (LPS). Binding of LF to LPS may result in neutralisation of LPS, which is hypothesized to play an important role in the immune regulatory role of LF [20]. In contrast, LF was shown to activate monocytes during DC differentiation, which resulted in diminished TLR activation [21]. Similarly, human LF was shown to induce the differentiation into anergic macrophages that were hyporesponsive towards TLR ligands [22]. However, LF binds LPS with a high binding affinity [23]. Since LPS induces differentiation of monocytes into tolerogenic DC as well [24], it is of interest to investigate the true immunomodulatory potential of LPS-free LF. A previous study showed that LPS-free LF induces the expression of pro-inflammatory cytokines on porcine derived macrophages in a TLR4-independent manner [25]. It is however unknown whether this TLR4-independent signaling of LF affects the functionality of human monocytes and dendritic cells. We therefore investigated whether LF, independently of bound LPS, is capable of inducing differentiation of human monocytes into tolerogenic dendritic cells.

## 2. Materials and Methods

### 2.1. Isolation of Bovine Lactoferrin

The whey fraction from bovine colostrum was collected after spinning the milk at 100,000 g for 45 min (Ultracentrifuge Avanti J301, Beckman Coulter, Brea, CA, USA). This casein and fat-free fraction was stored at −20 °C until further use. These whey proteins were thawed and diluted with washing buffer containing 0.01 M KH_2_PO_4_ and 0.1 M NaCl, pH 6.5. Samples were centrifuged at 23,500 *g* for 20 min and the supernatant was carefully collected through a filter paper to remove casein traces. Ion exchange chromatography (Akta Purifier, Pharmacia, Stockholm, Sweden) was used to isolate LF from the whey proteins. The Hiprep SP FF 16/10 column was preconditioned by rinsing the column with 100 mL washing buffer (3 mL/min; 12 mS/cm) and elution buffer (3 mL/min; 85 mS/cm). Whey proteins were loaded on the preconditioned column with a flow rate of 3 mL/min (HiLoad P50 pump, Pharmacia). After the complete volume of whey protein has run over the column, it was connected to the AKTA. Unbound matrix proteins were washed from the column with washing buffer (3 mL/min). Bovine lactoferrin was eluted from the column using an increasing gradient of 0.1–1 M NaCl. The preparation was desalted and concentrated on a 10 kDa Ultracel PLGC membrane using an Ultrafiltration Cell model 8200 (Amicon/Millipore).

### 2.2. Isolation and Culturing of Monocyte-Derived DC

Peripheral blood mononuclear cells (PBMCs) were isolated by density centrifugation from buffy coats obtained from healthy anonymous donors (Sanquin blood bank, Nijmegen, The Netherlands) as described previously [24]. A written informed consent was provided before blood collection. In short, 1:1 diluted blood in phosphate buffered saline (PBS) (Mg^2+^ and Ca^2+^ free, Lonza, BE17-516F, Basel, Switzerland) was loaded on Ficoll-Paque (Amersham Bioscience, Uppsala, Sweden) and centrifuged for 20 min at 500 g without brake. The PBMC layer was collected and washed three times with PBS. Cells were spun down and the pellet was resuspended with anti-human CD14 magnetic beads (BD Biosciences, 557769, Franklin Lakes, NJ, USA). CD14+ cells were isolated on a separation magnet (BD IMagnet, BD Biosciences) according to the manufacturer’s instructions. 100,000 monocytes per well were cultured in 96 cells wells flat bottom plates in RPMI 1640 (Gibco, Carlsbad, CA, USA, 22409-015) and 10% FCS (Gibco, 10270-106), normocin (100 µg/mL, Invivogen, anti-nr-1, San Diego, CA, USA), penicillin and streptomycin (100 U/mL, Gibco, 14150-122). Monocytes were differentiated into dendritic cells by culturing them for six days in the presence of 20 ng/mL IL-4 (Peptrotech; 200-04, Rocky Hill, CT, USA) and GM-CSF (Peprotech; 300-03, Rocky Hill, CT, USA) with or without 10 µg/mL, 250 µg/mL bovine lactoferrin (FC) or 10 nM VitD3 (Sigma-Aldrich, D1530, St. Louis, MO, USA). After six days, immature DC were matured with 1 µg/mL LPS (*Escherichia coli*, Sigma, L2880, St. Louis, MO, USA) or 3 µg/mL R848 (Invivogen: tlrl-r848-5) and 20 µg/mL Poly I:C (Sigma, P1530) for 48 h.

### 2.3. Isolation and Staining of moDC

After 6 days (immature-) or 8 days (mature-) moDC were incubated on ice (while shaking) for 30 min in ice cold FACS buffer (PBS (Lonza, BE17,516F) containing 0.5% BSA fraction V (Roche, 10735086002, Basel, Switzerland), 2.0 mM EDTA (Merck, 108418, Kenalworth, NJ, USA) and 0.05 NaN_3_) to facilitate the detachment of DC from the surface. Surface marker expression was analysed by using fluorochrome-conjugated antibodies directed against CD14 (FITC; BD Biosciences, 555397), CD86 (V450; BD Biosciences, 560357), CD83 (FITC; BD Biosciences, 556910), HLA-DR (APCef780; eBiosciences, 47-9956-42, San Diego, CA, USA), CD80 (PE-Cy5; BD Biosciences, 559370), PD-L1 (PE-Cy7; BD Biosciences, 558017), CD1a (PerCP/Cy5-5; Biolegend, 300130, San Diego, CA, USA). 10 µg/mL of human Fc Block (BD Bioscience, 554220) was added to the antibody mixture to block non-specific binding. Compensation beads (eBiosciences, 01-2222-41) stained with single antibodies were run for every experiment. Cells were washed with 200 µL FACS buffer and stained by incubating the antibody mixture for 30 min in the dark at 4 °C. Before measuring DRAQ7 (Abcam; ab109202, Cambridge, UK) was added and incubated for 10 min in the dark to stain nonviable cells. Cells were resuspended in 100 µL FACS buffer and acquired on a BD FACS Canto II (BD Biosciences) and analysed using the FlowJo software V10.

### 2.4. Quantification of Cytokine Levels in Supernatants

Levels of IL-8, IL-6, IL-10, TNF-α and IL-12p70 were measured in the supernatants of moDC cultures using cytometric bead array technique (BD Biosciences). Individual flex-sets for IL-8 (558277), IL-6 (558276), TNF-α (560112), IL-10 (558274) or IL-12p70 (558283) were run according to the manufacturer’s instructions.

### 2.5. Proteinase K Treatment and SDS-Page

LF was treated with 100 µg/mL proteinase K for 1 h at 46 °C followed by 10 min on 95 °C to inactivate the enzyme activity. 1 µg of LF and proteinase K treated LF were loaded on a SDS-page gel (Mini-PROTEAN TGX Precast SDS-page gel, Biorad, Berkeley, CA, USA) and run on 120 V for 1 h. The gel was stained with GelCode (Thermo Scientific, 24590, Waltham, MA, USA) according to the manufacturer’s instructions.

### 2.6. LPS Detection

LF and Triton X-114 treated LF was tested for LPS contamination by a recombinant factor C LAL assay that was performed according to the manufacturers recommendations (EndoZyme recombinant factor C assay, Hyglos; 609050, Bernried am Starnberger See, Germany).

### 2.7. Triton X-114 Treatment

LPS was removed from LF by an optimized Triton X-114 method (Amresco, cat. # M114, Solon, OH, USA) [26]. In short, 2% *v*/*v* Triton X-114 was added to the sample and the mixture was stirred for 30 min at 4 °C and thereafter transferred to a 41 °C water bath for 10 min. The micelles were spun down by centrifugation for 10 min at 20,000 *g* at 25 °C. The upper layer was collected and treated with 10 mg/mL Bio-beads SM-2 (cat. # 152-8920) under constant stirring at 4 °C to remove Triton X-114 traces.

### 2.8. TLR4 Reporter Assay

HEK-293 cells expressing human TLR4, CD14 and MD-2 and harbouring a pNIFTY construct (Invivogen, Toulouse, France) were grown on selective medium containing DMEM and Glutamax (Fisher Emergo, Landsmeer, The Netherlands) supplemented with 10% FCS, 100 µg/mL penicillin/streptomycin (Sigma, St. Louis, MO, USA), Zeozin (50 µg/mL), Normocin (100 µg/mL) and HygroGold (45 µg/mL) (Invitrogen, Carlsbad, CA, USA) in an atmosphere of 5% CO_2_ at 37 °C. HEK-293 cells were seeded at 3 × 10^5^ cells/mL and cultured overnight before stimulation the next day. NF-κB activation was measured after 24 h stimulation by adding Bright-Glo^TM^ (Promega, Fitchburg, MA, USA) substrate to cells. The plate was shaken and luminescence was measured using a spectramax M5 (Molecular Devices, Sunnyvale, CA, USA).

### 2.9. Statistics

Data was assessed for normality using a D’Agistino and Pearson omnibus test. A repeated measures ANOVA with Tukey’s multiple comparison test or Friedman test with Dunn’s multiple comparison post-hoc test was performed for normal and non-normal distributed data, respectively. Data is represented as mean ± standard error of the mean (SEM). Graphpad Prism V. 5.0 was used for all statistical analyses.

## 3. Results

**Figure 1:**
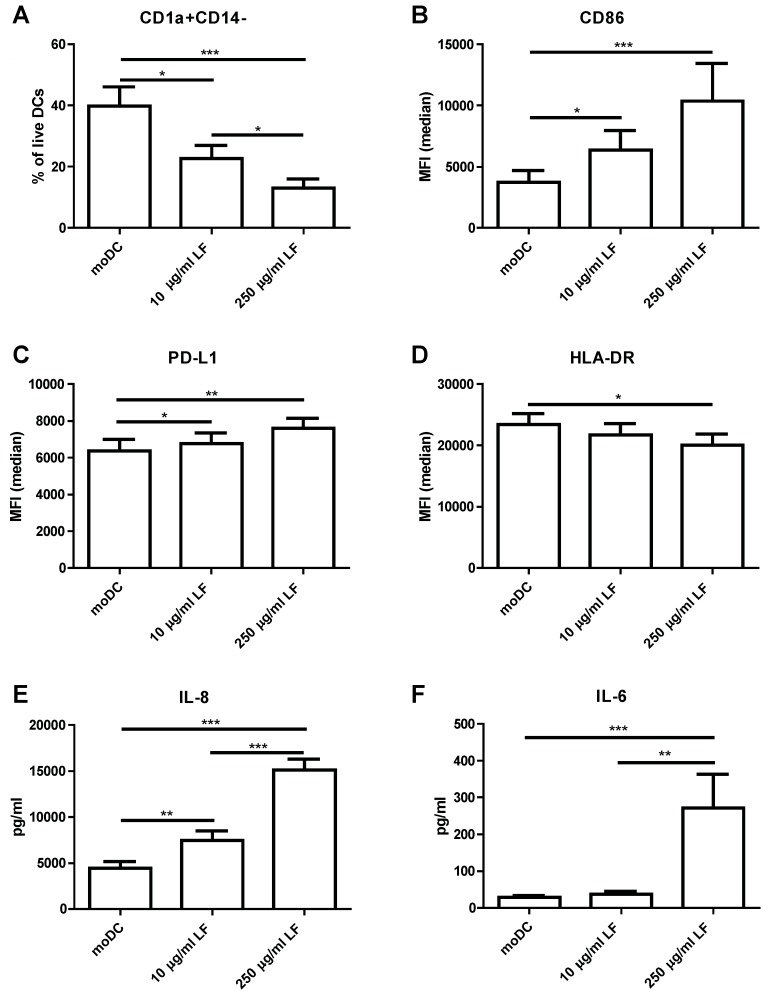
LF modulates DC differentiation. Monocytes were differentiated into moDC by culturing them for six days in the presence of IL-4 and GM-CSF with or without LF (10 or 250 µg/mL LF). (**A**) The percentage CD1a+CD14− DC and the median fluorescent intensity (MFI) of (**B**) CD86, (**C**) PD-L1 and (**D**) HLA-DR was shown. The production of (**E**) IL-8 and (**F**) IL-6 was measured in the supernatant by CBA. The mean ± SEM of four independent experiments with 12 different donors was shown. Significance is indicated by *** = *p* < 0.001, ** = *p* < 0.01 and * = *p* < 0.05.

Monocytes were differentiated into monocyte-derived DC (moDC) in the presence or absence of LF. Upon differentiation into moDC, monocytes lost CD14 expression and gained CD1a expression. Although LFDC were negative for CD14, a lower percentage of cells gained CD1a expression (Figure 1A). LFDC showed a higher expression of CD86 and PD-L1 (Figure 1B,C) and a significantly lower expression of HLA-DR when differentiated in the presence of 250 µg/mL LF (Figure 1D). These phenotypical changes induced by LF during differentiation were accompanied by a dose-dependent increase in IL-8 production (Figure 1E) and increase in IL-6 production when cultured in the presence of 250 µg/mL LF (Figure 1F). Interestingly, in contrast to the profound effects of LF during the differentiation of monocytes into moDC, LF did not induce phenotypic changes on moDC that were already differentiated (Figure S1). Moreover, moDC stimulated with LF or LF + Poly I:C and R848 for two days did not show phenotypic changes compared to moDC or moDC + Poly I:C and R848, respectively.

**Figure 2:**
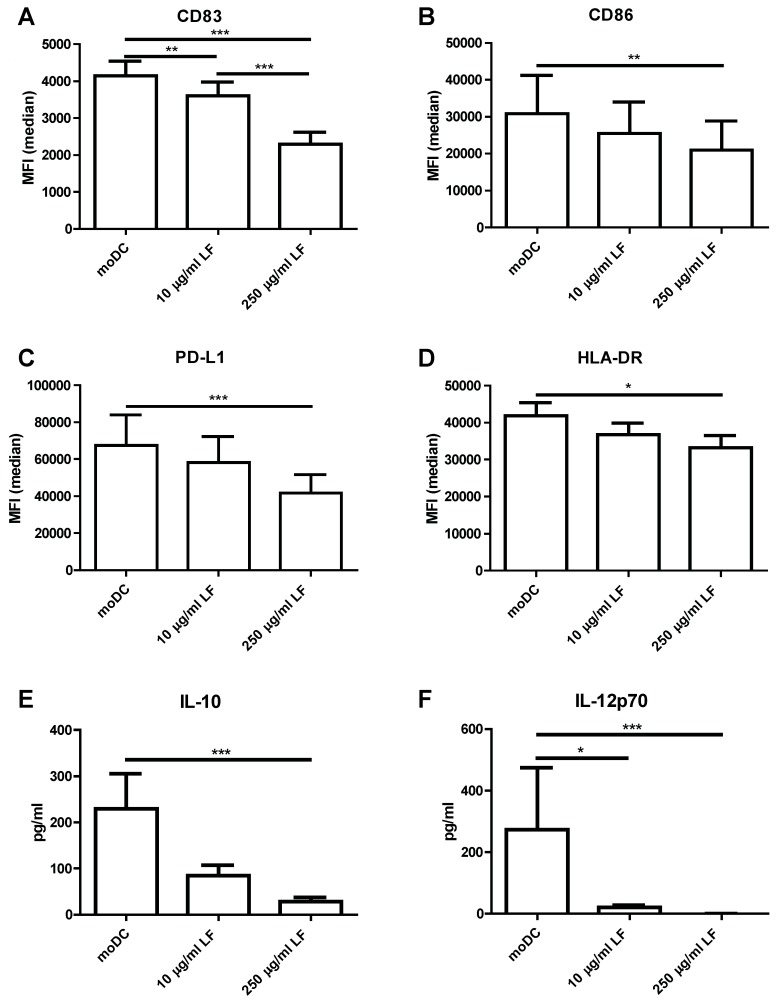
LFDC are hyporesponsive for LPS stimulation. Immature DC that were cultured in the presence or absence of LF were stimulated with 1 µg/mL LPS for 48 h. (**A**) The median fluorescent intensity (MFI) of (**A**) CD83, (**B**) CD86, (**C**) PD-L1 and (**D**) HLA-DR was shown. The production of (**E**) IL-10 and (**F**) IL-12p70 was measured in the supernatant by CBA. The mean ± SEM of four independent experiments with 12 different donors was shown. Significance is indicated by *** = *p* < 0.001, ** = *p* < 0.01 and * = *p* < 0.05.

Next, we investigated the responsiveness of LFDC by stimulating the cells with LPS. LFDC showed to be hyporesponsive towards LPS as observed by the reduced induction of the maturation marker CD83 (Figure 2A) and costimulatory molecules CD86 (Figure 2B), PD-L1 (Figure 2C) and CD80 (Figure S2A) upon LPS stimulation. HLA-DR expression was lower on mature LFDC compared to mature moDC (Figure 2D). In line with their phenotype, LFDC also produced lower cytokine levels, showing a lower production of IL-10 (Figure 2E), IL-6 (Figure S2B), TNF (Figure S2C) and abrogated levels of IL-12p70 (Figure 2F). Since DC were differentiated and subsequently stimulated in the presence of LF, we wanted to exclude the possibly that the hyporesponsiveness towards LPS was caused by neutralisation of LPS by LF. We showed that the surface marker expression was unaffected by replacing ¾ of the medium and that the cells were also hyporesponsive towards R848 and Poly:IC stimulation, indicating that the effect was not mediated by LPS neutralisation (Figure S3).

**Figure 3:**
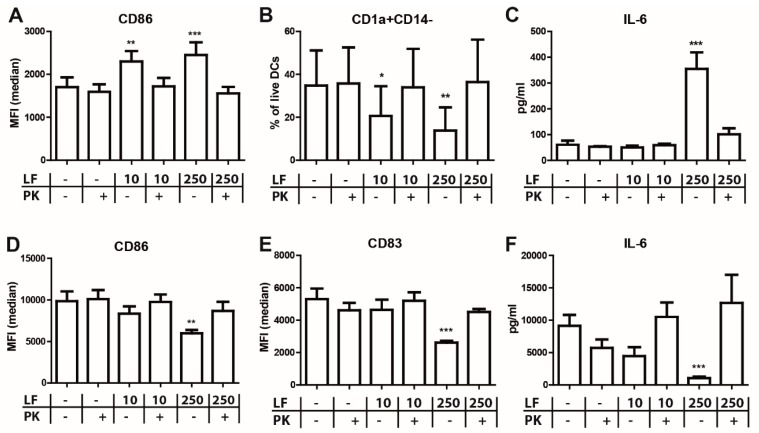
Proteinase K treatment of LF restores responsiveness towards LPS. (**A**–**C**) monocytes were cultured in the presence of IL-4 and GM-CSF with or without LF or proteinase K treated LF. (**D**,**E**) These immature DC were subsequently stimulated with 1 µg/mL LPS for 48 h. The median fluorescent intensity (MFI) of (**A**,**D**) CD86 and (E) CD83 and (**B**) the percentage CD1a+CD14− DC was shown. (**C**,**F**) The production IL-6 was measured in the supernatant by CBA. The mean ± SEM of 3 different donors was shown. Significance is indicated by *** = *p* < 0.001, ** = *p* < 0.01 and * = *p* < 0.05.

LF is reported to bind LPS and has been postulated to induce immunomodulation via TLR4-dependent mechanisms. However, previously we showed that low concentrations of LPS can also LPS induce these phenotypic changes. We therefore measured the concentration of LPS in LF by an endozyme LAL assay. LF used in this study showed concentrations of 2.6 EU LPS/mg LF (Figure S4A). We therefore applied an optimized Triton X-114 method [26] to reduce LPS levels. This method reduced the endotoxin levels to 0.58 EU LPS/mg LF (Figure S4A). Despite this five-fold decrease in LPS levels, the immunomodulatory activity of LF remained the same (Figure S4). Nevertheless, we have previously shown that low concentrations of LPS induce endotoxin tolerance [24]. We therefore wanted to be absolutely certain that the functional effects of LF were not mediated by LPS. Therefore, we treated LF with proteinase K to degrade the protein and release any potentially bound endotoxins. We confirmed that LF was completely degraded after proteinase K treatment as observed on SDS-page gel (Figure S5A), Additionally, we showed by using a TLR4 reporter assay that the NF-κB inducing capacity of LPS was unaltered by proteinase K treatment (Figure S5B), indicating that the LPS released from bound to LF remains functional. Interestingly, the dose-dependent increase of CD86 (Figure 3A) and PD-L1 (Figure S6B) and decreased CD1a (Figure 3B) expression induced by LF was reduced to moDC levels after proteinase K treatment. Similarly, the LF induced production of IL-6 (Figure 3C) and IL-8 (Figure S6A) during DC differentiation was abrogated after degradation of the protein. Thus, the LF-induced phenotype on immature DC was completely abolished by proteinase K treatment of the protein, showing that the effect is mediated by LF and not by LPS. In line with these findings, the responsiveness towards LPS of LFDC was completely restored to that of moDC upon proteinase K treatment as shown by the expression of CD86 (Figure 3D), CD83 (Figure 3E), CD80 and PD-L1 (Figure S7A,B) and the production of cytokines such as IL-6 (Figure 3F), IL-12p70, TNF and IL-10 (Figure S7D–F).

## 4. Discussion

In this paper, we show that LF modulates human DC differentiation and function. Additionally, we show that its immunomodulatory capacity is not mediated by LPS. With this study, we confirm and expand on the study of Puddu et al. (2011), that showed that LF is capable of inducing differentiation into tolerogenic DC [21]. Since trace amounts of LPS can already induce a tolerogenic phenotype [24], it is necessary to assess that LF and not LPS is responsible for the effect. Here, we show that LF alters the differentiation of monocytes into DC, resulting in a phenotypical distinct DC type which is hyporesponsive towards several TLR ligands.

It has been proposed that the GI tract is in a constant state of low-grade inflammation or “primed homeostasis” due to the constant exposure to commensal bacteria and microbial products in which monocytes are recruited toward the GI tract [27,28]. In line with this thought, a local micro-environment comprised of dietary and microbial factors may steer monocyte differentiation. We therefore investigated the effect of LF on monocyte differentiation into DC. It is well established that monocytes lose CD14 expression and gain CD1a expression during differentiation into moDC. In the presence of LF, DC lose CD14 expression and gain less CD1a expression compared to conventional differentiated moDC. CD1a is an important functional marker for immature DC. Moreover, CD1a+ DC produce higher IL-12p70 levels and lower IL-10 levels upon stimulation and show a lower internalisation capacity compared to CD1a-DC [29,30]. Potential explanations for the reduced CD1a expression on LFDC can be induction of PPARγ activity [30], differentiation into more macrophage-like cells due to IL-6-mediated autocrine M-CSF production [31], or inhibition of GM-CSF signalling [32]. Interestingly, an embryonic fibroblast cell line transfected with the human lactoferrin gene showed inhibited GM-CSF levels upon stimulation [33]. LF is, in contrast to moDC, internalised into the nucleus of human monocytes [21]. This finding makes it appealing to speculate about direct effect of internalised LF on the GM-CSF promotor in monocytes. Additionally, LF induces the production of IL-6 and IL-8 within 24 h of differentiation (data not shown). Although IL-6 has been shown to be an important cytokine capable of modulating DC differentiation [18] and boosting autocrine G-CSF production [31], Puddu et al. (2011) showed that the effects of LF are not mediated by IL-6. In line with these findings we show that IL-6 production was not elevated if monocytes are differentiated in the presence of 10 µg/mL LF and yet this concentration is sufficient to induce DC that are hyporesponsive towards TLR ligands.

LFDC produced much lower cytokine levels and upregulated costimulatory molecules markers to a lesser extent compared to moDC upon contact with TLR ligands. Moreover, they showed a semi-mature (CD83^int^CD86^int^) phenotype, which is postulated to induce polarisation of naive T cells in regulatory T cells [34]. Thus, LF induces the differentiation of monocytes into more tolerogenic DC. In contrast to the effect of LF on monocytes, we show that LF does not induce phenotypical changes on human moDC nor modulate Poly I:C and R848-induced inflammation. These findings are in line with previous research, showing internalisation of LF into the nucleus of human monocytes and not in moDC [21]. Their findings propose that the uptake of LF is mediated by a receptor expressed on human monocytes that is not expressed on moDC, which could explain the distinct effect of LF on both cell types. LF binds to intelectin-1 (i.e., intestinal lactoferrin receptor), which is expressed on epithelial cells [19]. To date, no evidence suggests expression of intelectin-1 on myeloid cells. LF also binds DC-SIGN which is expressed on monocytes and moDC [18]. However, the observed effects are also not likely mediated via DC-SIGN since its expression increases upon differentiation into DC [35]. Additionally, it has been suggested that LF binds with low affinity to several other receptors (e.g., RAGE and MNR). Interestingly, human LF was also shown to bind to soluble CD14 [36]. Since CD14 expression is lost upon differentiation of monocyte into moDC, it is tempting to speculate that our phenotypic changes are induced via CD14. However, LF in concentration used in this study did not activate NF-κB on CD14-MD-2:TLR4 expressing HEK cells (data not shown). Recently, CD14 was shown to be essential for endocytosis of the TLR4 complex [37], showing that its function is more than just facilitating LPS to bind TLR4. We therefore hypothesize that LF:LPS complexes can be internalized on monocytes in a unknown CD14-mediated manner.

This study shows, in line with the literature, that bovine LF, as well as human LF, induces differentiation of monocytes into hyporesponsive DC [21] and macrophages [22], respectively. However, due to the high binding affinity of LF to LPS, it is essential to investigate whether LPS or LF is responsible for this tolerogenic phenotype. Importantly, we showed that our sample did contain traces of LPS as measured by a LAL assay. In this assay, a recombinant factor C protein is used to detect LPS, which binds the lipid A part of the molecule [38]. LF was also shown to bind the lipid A part of the molecule [23], which could shield LPS from binding TLR4 and apparently not factor C. According to the LPS contamination measured in our sample (i.e., 2.6 EU/mg), the concentrations of LF used in our study of 0.25 mg/mL and 0.01 mg/mL, thus, contain 0.65 EU and 0.026 EU, respectively. We have shown that LPS contamination of >0.5 EU induces the differentiation into tolerogenic DC [24]. The concentrations of LPS measured in LF in this study could, thus, theoretically explain the tolerogenic DC phenotype. Nevertheless, we demonstrated that the effects of LF are lost upon proteinase K treatment and that Triton X-114-treated LF shows the same immunomodulatory capacity compared to non-treated LF. We thereby exclude the possibility that effects of LF are mediated by endotoxin tolerance. This finding is in line with earlier research showing that the induced expression of pro-inflammatory genes in porcine macrophages is mediated in a TLR4-independent manner [25].

This study adds on to the current understanding of the role of LF in immune regulation. Additionally, LF is well-known for its anti-pathogenic activity. Hence, LF supplementation to infants and immunocompromised individuals has been studied to investigate its efficacy against inflammatory conditions. Moreover, several clinical studies in children have been conducted with LF, all showing no adverse effects of LF supplementation [39]. LF supplementation to breast milk or infant formulas has been shown to reduce the incidence of sepsis VLWB infants [3,4,5,6,7]. These VLWB infants often suffer from excessive gut inflammation and have an impaired epithelial barrier functioning, which may result in necrotizing enterocolitis [40]. Additionally, several studies show that LF fortification to early life nutrition may alleviate symptoms of viral infections [41,42,43,44]. Moreover, LF supplementation to children <5 years of age and to infants in the first year of life showed a reduction of the incidence of rotaviral gastroenteritis [41] and lower respiratory tract infections [42]. Similarly, LF supplementation to infant nutrition resulted in a lower incidence of symptoms of respiratory illness (e.g., running nose, coughing, wheezing) compared to infants receiving non-fortified formula [43]. LF supplementation has also been investigated for its additive effect in HIV therapy in children by measuring several immune parameters and viral titers. Interestingly, phagocytic activity, CD14/TLR2 expression and IL-12p70/IL-10 ratio in CD14+ was increased in children receiving LF supplementation [44]. Thus, apart from functioning as a direct anti-pathogenic protein, LF may inhibit infections by its immunomodulatory capacity. Since, LF is poorly digested in the GI tract of infants and a fraction of the protein is taken up intact and reaches the circulation [9,12,13], it may impact the functionality of monocytes and DC in vivo*.* Larger cohorts should validate the protective effect of LF supplementation against viral infections and sepsis and investigate its immunomodulatory potential in vivo.

## 5. Conclusions

Taken together, our results show that LF inhibits DC differentiation which hampers their responsiveness towards TLR ligands. Additionally, we showed that these effects are diminished after degrading the protein, formally showing that the LF-induced differentiation of monocytes into hyporesponsive DC is not mediated by endotoxin tolerance. This study indicates that LF may promote immune homeostasis in the gastrointestinal tract.

## Supplementary Materials

The following are available online at http://www.mdpi.com/2072-6643/10/7/848/s1. Figure S1: LF does not modulate immature DC activation. Monocytes were differentiated into moDC by culturing them for six days in the presence of IL-4 and GM-CSF. These immature DC were stimulated with 20 µg/mL Poly I:C and 3 µg/mL R848 in the presence or absence of LF. The median fluorescent intensity (MFI) of (A) CD8, (B) CD86, (D) HLA-DR, (E) PD-L1, (F) CD80 and the percentage of CD83 + CD86 + DC was shown. The expression of three individual donors with mean and standard deviation was shown in a scatter plot. Figure S2: LFDC are hyporesponsive for LPS stimulation. Immature DC that were cultured in the presence or absence of LF were stimulated with 1 µg/mL LPS for 48 h. (A) The median fluorescent intensity (MFI) of (A) CD80 was shown. The production of (B) IL-6 and (C) TNF was measured in the supernatant by CBA. The mean ± SEM of four independent experiments with 12 different donors was shown. Significance is indicated by *** = *p <* 0.001, ** = *p* < 0.01 and * = *p <* 0.05. Figure S3: hyporesponsiveness of LFDC is not mediated by decoy activity. Immature DC that were cultured in the presence or absence of LF were stimulated with 1 µg/mL LPS (A)without or (B) with replacing ¾ of the medium or (C) 20 µg/mL Poly I:C and 3 µg/mL R848 for 48 h. (A) The median fluorescent intensity (MFI) of (A) CD80 was shown. The mean ± SEM 3 different donors was shown. Figure S4: DC modulatory activity of LF is not reduced by Triton X-114 treatment. (A) an Endozyme LAL assay was used to detect LPS in LF before or after applying an optimised Triton X-114 method. Immature DC were cultured in the presence or absence of Triton X-114 treated or non-treated LF. (A) The percentage CD1a+ CD14− DC was shown on immature DC. These immature DC were stimulated with 1 µg/mL LPS for 48 h. Median fluorescent intensity (MFI) of (C) CD83 and (D) CD86 was shown. The production of (E) IL-10 and (F) IL-12p70 was measured in the supernatant by CBA. The mean ± SEM of 3 different donors was shown. Significance is indicated by *** = *p* < 0.001, ** = *p* < 0.01 and * = *p* < 0.05. Figure S5: Proteinase K treatment does not affect NF-κB activation via TLR4 by LPS. (A) 1 µg/mL LF was loaded before and after proteinase K treatment on SDS-PAGE gel. (B) LPS, proteinase K treated LPS and heated LPS was tested for its NF-κB activation in a TLR4 reporter assay. Figure S6: proteinase K treatment of LF abrogates its effect on DC differentiation. Monocytes were cultured in the presence of IL-4 and GM-CSF with or without LF or proteinase K treated LF. (A) The production of IL-8 was measured in the supernatant by CBA. The median fluorescent intensity (MFI) of (B) PD-L1 and (C) HLA-DR was shown. The mean ± SEM of 3 different donors was shown. Figure S7: proteinase K treatment of LF restores responsiveness towards LPS. Monocytes were cultured in the presence of IL-4 and GM-CSF with or without LF or proteinase K treated LF for six days and subsequently stimulated with 1 µg/mL LPS for 48 h. The median fluorescent intensity (MFI) of (A) CD80, (B) PD-L1 and (C) HLA-DR was shown. The production of (D) TNF, (E) IL-10 and (F) IL-12p70 was measured in the supernatant by CBA. The mean ± SEM of 3 different donors was shown. Significance is indicated by *** = *p* < 0.001, ** = *p* < 0.01 and * = *p* < 0.05.
