# Supplementary material for: Bovine Lactoferrin Modulates Dendritic Cell Differentiation and Function
Source: Nutrients. 2018 Jun 29;10(7):848. doi: 10.3390/nu10070848 (PMC6073808; doi:10.3390/nu10070848)
Supplement: Supplementary file 1 [file nutrients-10-00848-s001.pdf]

Supplementary materials: Bovine Lactoferrin Modulates Dendritic Cell Differentiation and Function

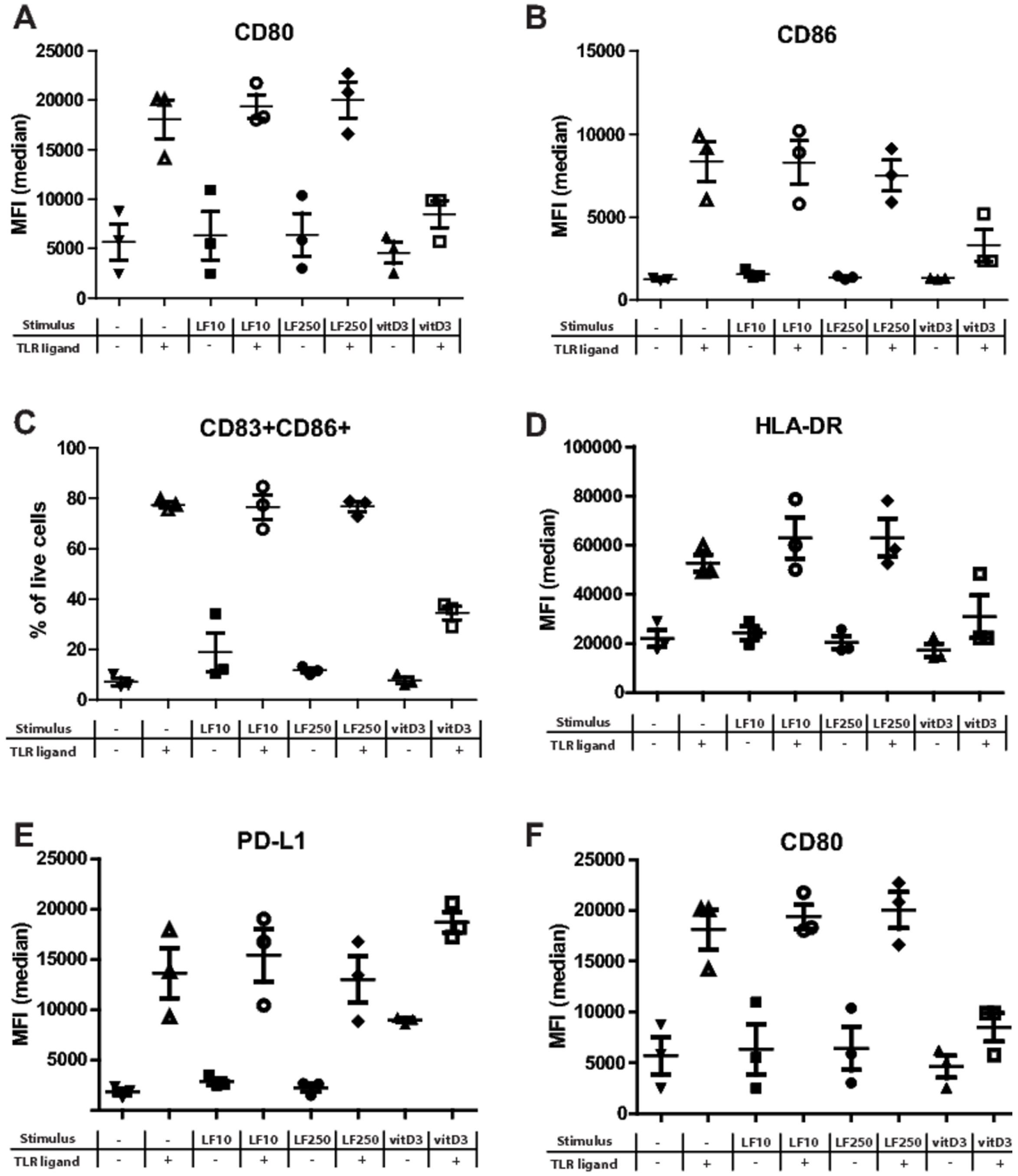

Figure S1. LF does not modulate immature DC activation. Monocytes were differentiated into moDC by culturing them for six days in the presence of IL-4 and GM-CSF. These immature DC were stimulated with 20 μg/mL Poly I:C and 3 μg/mL R848 in the presence or absence of LF. The median fluorescent intensity (MFI) of (**A**) CD8, (**B**) CD86, (**D**) HLA-DR, (**E**) PD-L1, (**F**) CD80 and the percentage of CD83 + CD86 + DC was shown. The expression of three individual donors with mean and standard deviation was shown in a scatter plot.

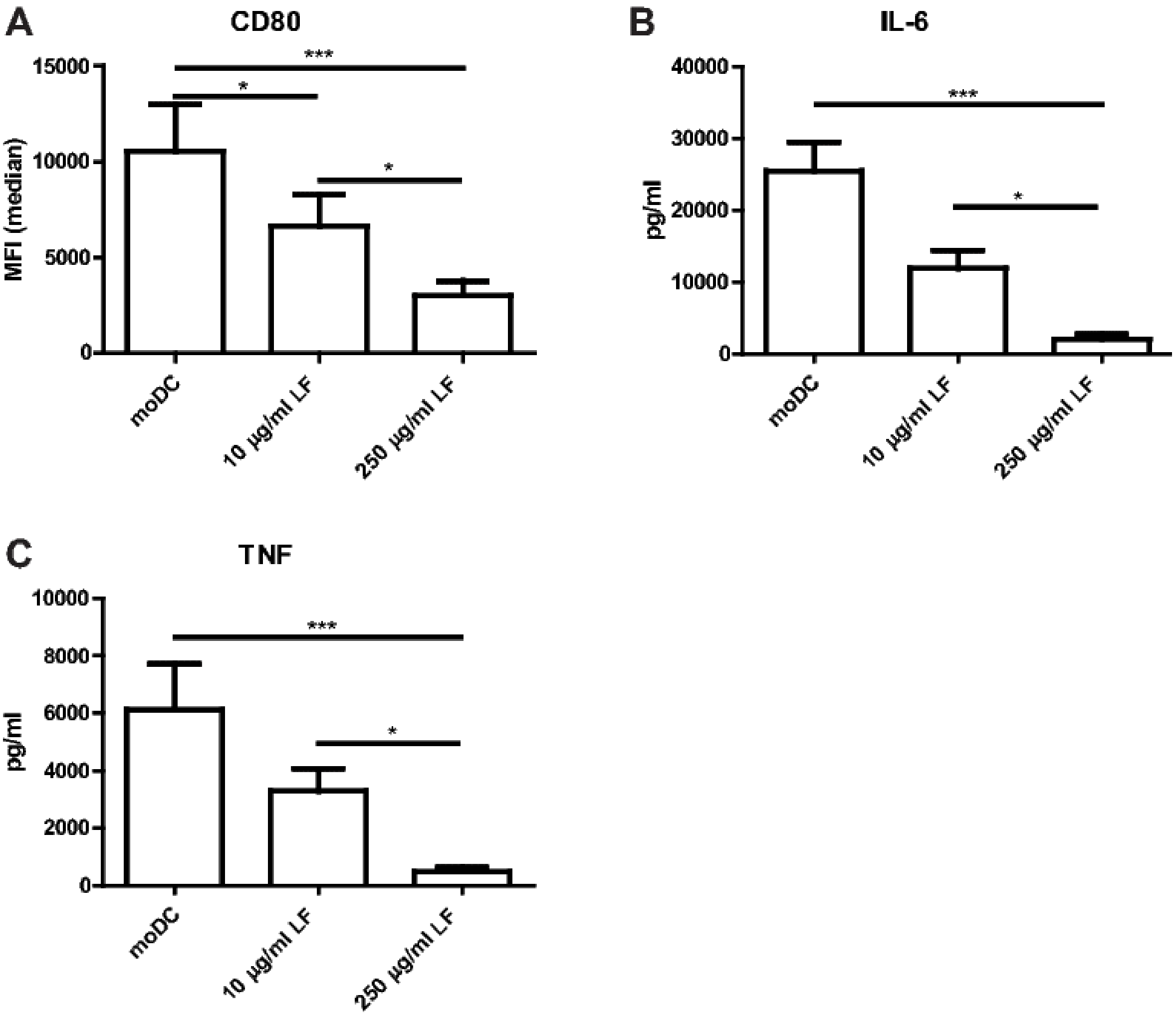

Figure S2. LFDC are hyporesponsive for LPS stimulation. Immature DC that were cultured in the presence or absence of LF were stimulated with 1 µg/mL LPS for 48 h. (**A**) The median fluorescent intensity (MFI) of (**A**) CD80 was shown. The production of (**B**) IL-6 and (**C**) TNF was measured in the supernatant by CBA. The mean ± SEM of four independent experiments with 12 different donors was shown. Significance is indicated by *** = p <0.001, ** = p <0.01 and * = p <0.05.

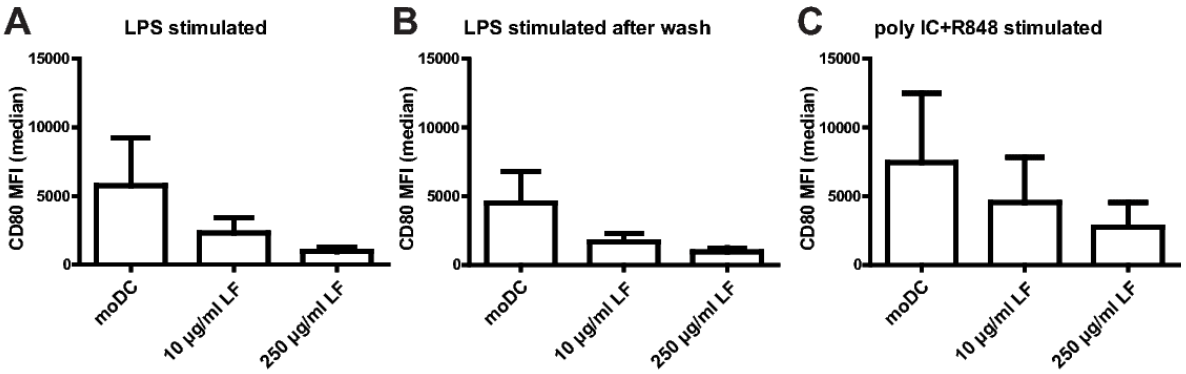

Figure S3. hyporesponsiveness of LFDC is not mediated by decoy activity. Immature DC that were cultured in the presence or absence of LF were stimulated with 1 µg/mL LPS (**A**)without or (**B**) with replacing ¾ of the medium or (**C**) 20 µg/mL Poly I:C and 3 µg/mL R848 for 48 h. (**A**) The median fluorescent intensity (MFI) of (**A**) CD80 was shown. The mean ± SEM 3 different donors was shown.

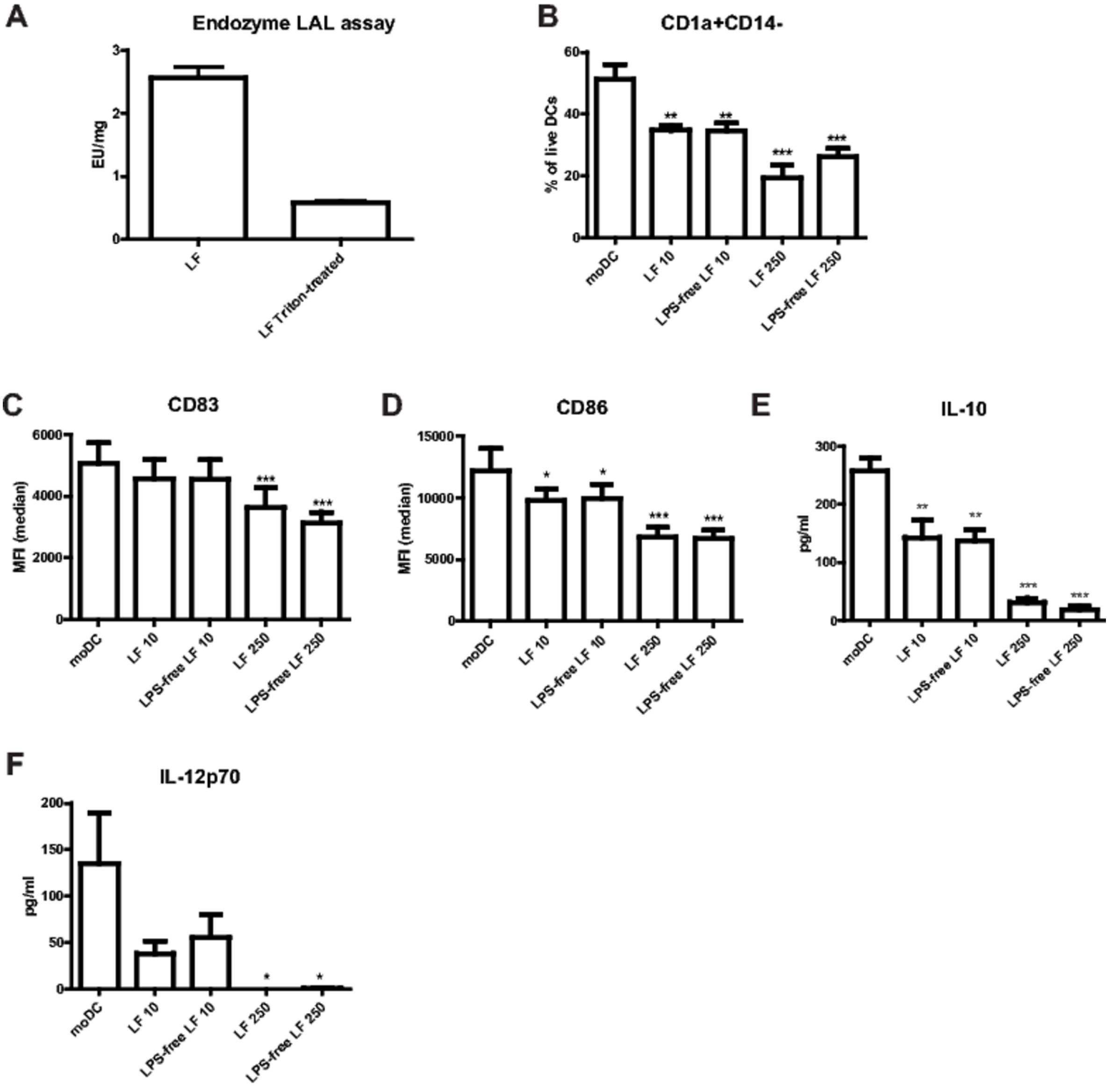

Figure S4. DC modulatory activity of LF is not reduced by Triton X-114 treatment. (**A**) an Endozyme LAL assay was used to detect LPS in LF before or after applying an optimised Triton X-114 method. Immature DC were cultured in the presence or absence of Triton X-114 treated or non-treated LF. (**A**) The percentage CD1a+ CD14− DC was shown on immature DC. These immature DC were stimulated with 1 µg/mL LPS for 48 h. Median fluorescent intensity (MFI) of (**C**) CD83 and (**D**) CD86 was shown. The production of (**E**) IL-10 and (**F**) IL-12p70 was measured in the supernatant by CBA. The mean ± SEM of 3 different donors was shown. Significance is indicated by *** = p <0.001, ** = p <0.01 and * = p <0.05.

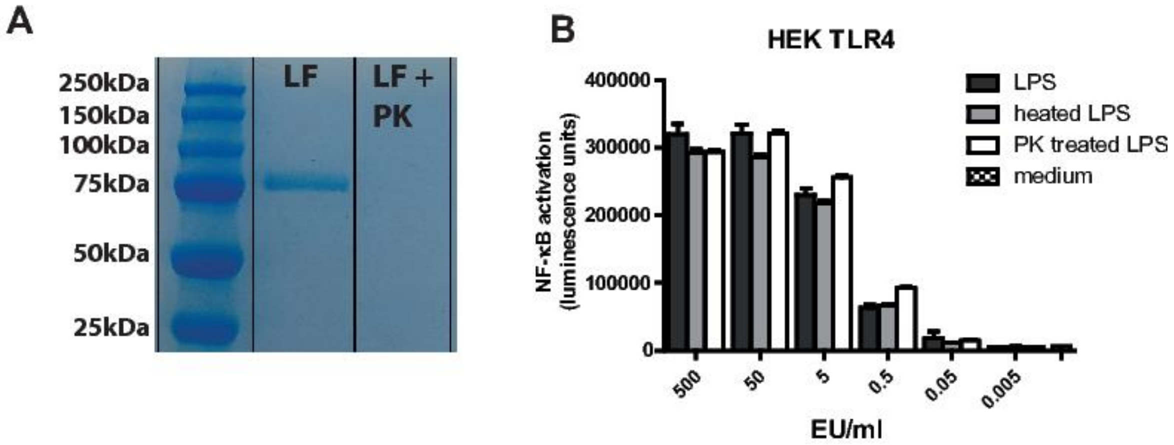

Figure S5. Proteinase K treatment does not affect NF-κB activation via TLR4 by LPS. (**A**) 1 μg/mL LF was loaded before and after proteinase K treatment on SDS-PAGE gel. (**B**) LPS, proteinase K treated LPS and heated LPS was tested for its NF-κB activation in a TLR4 reporter assay.

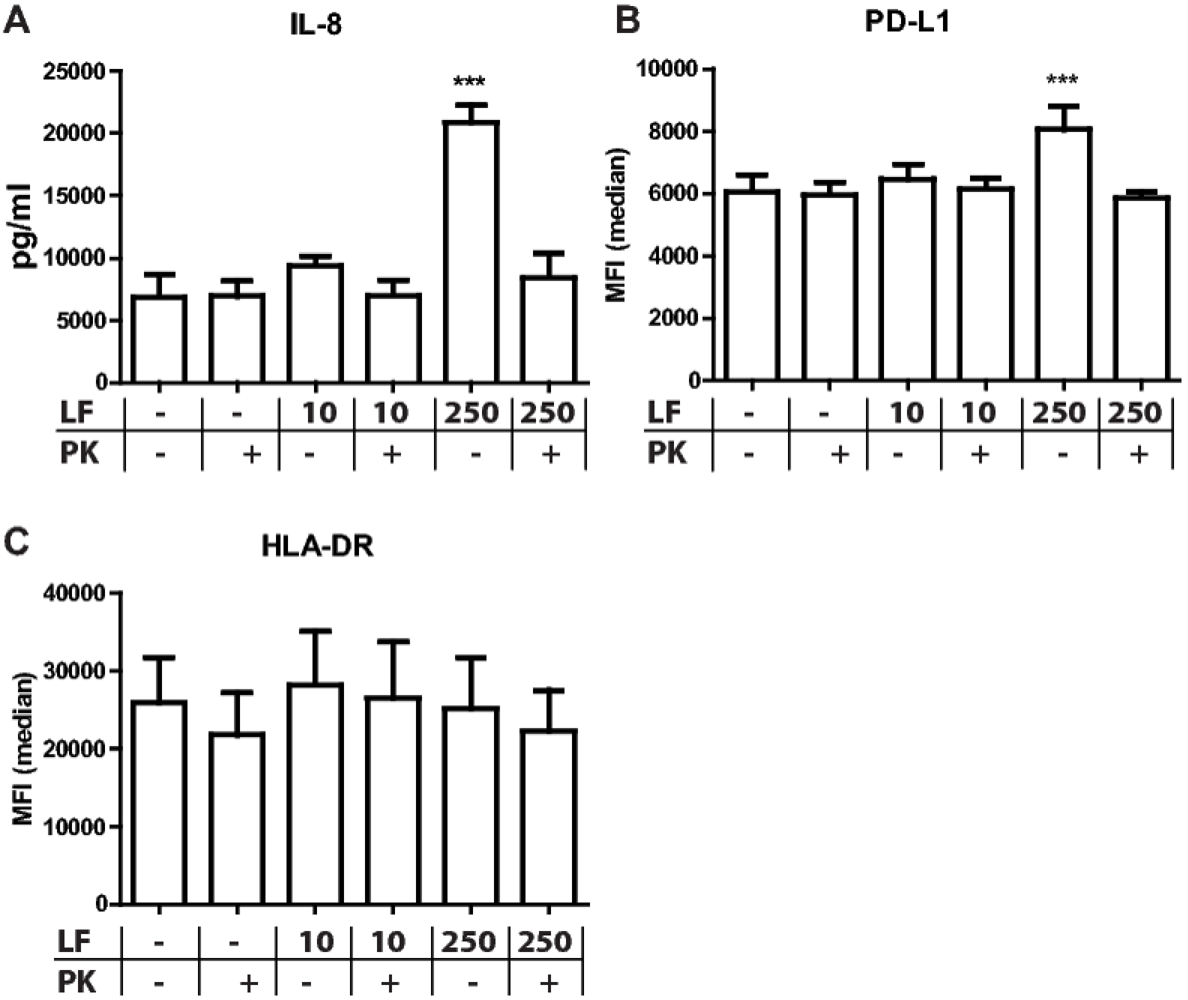

Figure S6. proteinase K treatment of LF abrogates its effect on DC differentiation. Monocytes were cultured in the presence of IL-4 and GM-CSF with or without LF or proteinase K treated LF. (**A**) The production of IL-8 was measured in the supernatant by CBA. The median fluorescent intensity (MFI) of (**B**) PD-L1 and (**C**) HLA-DR was shown. The mean ± SEM of 3 different donors was shown.

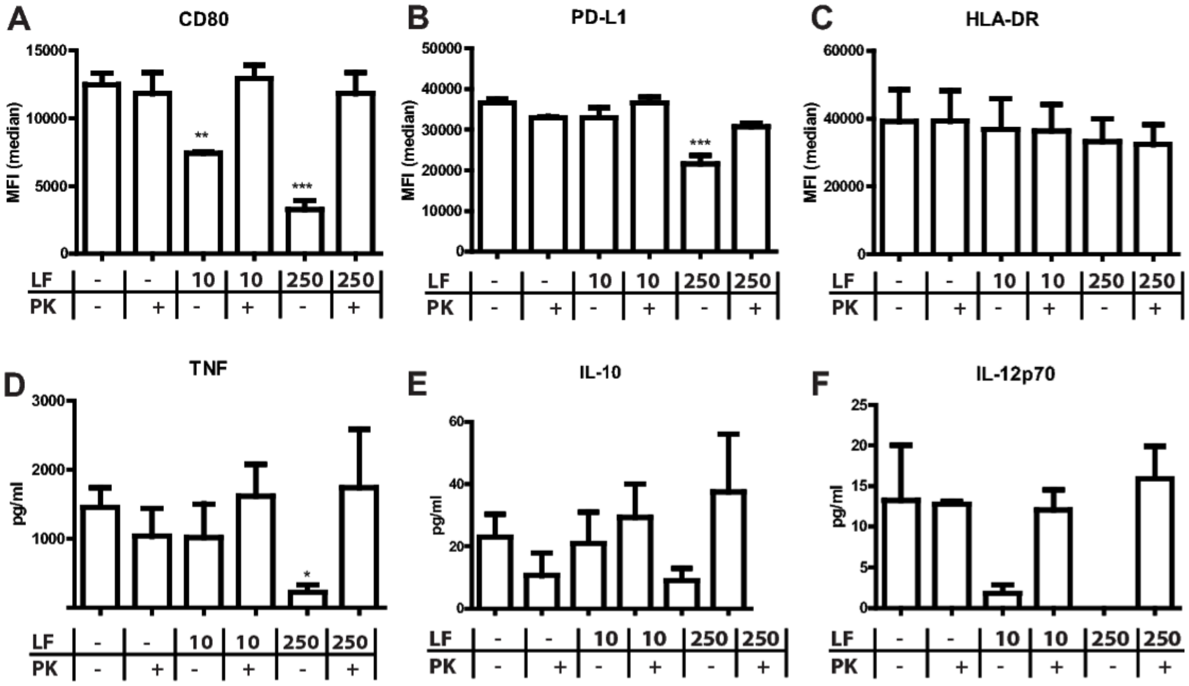

Figure S7. proteinase K treatment of LF restores responsiveness towards LPS. Monocytes were cultured in the presence of IL-4 and GM-CSF with or without LF or proteinase K treated LF for six days and subsequently stimulated with 1 µg/mL LPS for 48 h. The median fluorescent intensity (MFI) of (**A**) CD80, (**B**) PD-L1 and (**C**) HLA-DR was shown. The production of (**D**) TNF, (**E**) IL-10 and (**F**) IL-12p70 was measured in the supernatant by CBA. The mean ± SEM of 3 different donors was shown. Significance is indicated by *** = p <0.001, ** = p <0.01 and * = p <0.05.
